# Supplementary material for: Distribution of human papillomavirus genotypes by severity of cervical lesions in HPV screened positive women from the ESTAMPA study in Latin America
Source: PLoS One. 2022 Jul 29;17(7):e0272205. doi: 10.1371/journal.pone.0272205 (PMC9337688; doi:10.1371/journal.pone.0272205)
Supplement: S1 Table — Number of positive participants, prevalence (%) and 95% confidence intervals for each HPV risk-based group shown within each histological diagnosis. Genotypes included in five risk-based groups as follows: (1) HPV16/18 (carcinogenic types), (2) other HR-HPV (including other carcinogenic HPV31/33/35/39/45/51/52/56/58/59 types, probably carcinogenic HPV68 type and possibly carcinogenic HPV66 type; all twelve of them in the HPV screening techniques cocktails); (3) possibly HR-HPV (including other possibly carcinogenic HPV26/ 34/53/69/70/73/82 types), (4) LR-HPV (including low-risk HPV6/11/40/42/43/44/54/55/57/61/71/72/81/83/84/89 types) and (5) negative (none of the genotypes detected). Participants assigned to one single risk-based group considering a hierarchy from HPV16/18, other HR-HPV, possibly HR-HPV to LR-HPV in relation to the positivity of the corresponding genotypes. P-value from a trend test for proportions to assess the association of the prevalence with the histological grade for each HPV risk-based groups shown. (DOCX) [file pone.0272205.s001.docx]

|  | Colposcopy/biopsy Neg  n (% 95%IC) | CIN1  n (% 95%IC) | | CIN2  n (% 95%IC) | CIN3  n (% 95%IC) | Cancer  n (% 95%IC) |  |
| --- | --- | --- | --- | --- | --- | --- | --- |
|  |  |  | |  |  |  |  |
| HPV16/18 | 122 (20.6% 17.4-24.1) | 49 (18.8% 14.2-24) | | 37 (30.6% 22.5-39.6) | 110 (56.7% 49.4-63.8) | 59 (71.1% 60.1-80.5) | p<0.001 |
| Other HR-HPV | 350 (59% 54.9-63) | 145 (55.6% 49.3-61.7) | | 62 (51.2% 42-60.4) | 75 (38.7% 31.8-45.9) | 22 (26.5% 17.4-37.3) | p<0.001 |
| Possibly HR-HPV | 20 (3.4% 2.1-5.2) | 15 (5.7% 3.3-9.3) | | 9 (7.4% 3.5-13.7) | 3 (1.5% 0.3-4.5) | 1 (1.2% 0-6.5) | p=0.36 |
| LR-HPV | 18 (3% 1.8-4.8) | 9 (3.4% 1.6-6.4) | | 7 (5.8% 2.4-11.6) | 0 (0% 0-1.9) | 1 (1.2% 0-6.5) | p=0.1 |
| Negative | 83 (14% 11.3-17.1) | 43 (16.5% 12.2-21.5) | | 6 (5% 1.8-10.5) | 6 (3.1% 1.1-6.6) | 0 (0% 0-4.3) | p<0.001 |
|  | 593 (100%) | 261 (100%) | | 121 (100%) | 191 (100%) | 83 (100%) |  |
|  |  |  |  | |  |  |  |

**Table S1**. **Prevalence of HPV genotype risk-based groups within histological diagnoses in HPV screened positive women.** Number of positive participants, prevalence (%) and 95% confidence intervals for each HPV risk-based group shown within each histological diagnosis. Genotypes included in five risk-based groups as follows: (1) HPV16/18 (carcinogenic types), (2) other HR-HPV (including other carcinogenic HPV31/33/35/39/45/51/52/56/58/59 types, probably carcinogenic HPV68 type and possibly carcinogenic HPV66 type; all twelve of them in the HPV screening techniques cocktails); (3) possibly HR-HPV (including other possibly carcinogenic HPV26/ 34/53/69/70/73/82 types), (4) LR-HPV (including low-risk HPV6/11/40/42/43/44/54/55/57/61/71/72/81/83/84/89 types) and (5) negative (none of the genotypes detected). Participants assigned to one single risk-based group considering a hierarchy from HPV16/18, other HR-HPV, possibly HR-HPV to LR-HPV in relation to the positivity of the corresponding genotypes. P-value from a trend test for proportions to assess the association of the prevalence with the histological grade for each HPV risk-based groups shown.
