# Supplementary material for: Distribution of human papillomavirus genotypes by severity of cervical lesions in HPV screened positive women from the ESTAMPA study in Latin America
Source: PLoS One. 2022 Jul 29;17(7):e0272205. doi: 10.1371/journal.pone.0272205 (PMC9337688; doi:10.1371/journal.pone.0272205)
Supplement: S2 Table — Number of positive participants, prevalence (%) and 95% confidence intervals shown within each histological group. Results shown for individual HPV genotypes (irrespective of positivity for other genotypes). (DOCX) [file pone.0272205.s002.docx]

|  | ≤CIN1 n=854  n (% 95%IC) | CIN2 n=121  n (% 95%IC) | CIN3 n=194  n (% 95%IC) | Cancer n=83  n (% 95%IC) |
| --- | --- | --- | --- | --- |
| Possibly HR-HPV genotypes |  |  |  |  |
| HPV26 | 3 (0.4% 0.1-1) | 2 (1.7% 0.2-5.8) | 0 (0% 0-1.9) | 0 (0% 0-4.3) |
| HPV34 | 6 (0.7% 0.3-1.5) | 0 (0% 0-3) | 0 (0% 0-1.9) | 0 (0% 0-4.3) |
| HPV53 | 52 (6.1% 4.6-7.9) | 6 (5% 1.8-10.5) | 10 (5.2% 2.5-9.3) | 0 (0% 0-4.3) |
| HPV69 | 2 (0.2% 0-0.8) | 2 (1.7% 0.2-5.8) | 1 (0.5% 0-2.8) | 0 (0% 0-4.3) |
| HPV70 | 28 (3.3% 2.2-4.7) | 4 (3.3% 0.9-8.2) | 3 (1.5% 0.3-4.5) | 1 (1.2% 0-6.5) |
| HPV73 | 20 (2.3% 1.4-3.6) | 3 (2.5% 0.5-7.1) | 1 (0.5% 0-2.8) | 2 (2.4% 0.3-8.4) |
| HPV82 | 17 (2% 1.2-3.2) | 3 (2.5% 0.5-7.1) | 3 (1.5% 0.3-4.5) | 0 (0% 0-4.3) |
|  |  |  |  |  |
| LR-HPV genotypes |  |  |  |  |
| HPV6 | 21 (2.5% 1.5-3.7) | 2 (1.7% 0.2-5.8) | 3 (1.5% 0.3-4.5) | 0 (0% 0-4.3) |
| HPV11 | 5 (0.6% 0.2-1.4) | 2 (1.7% 0.2-5.8) | 0 (0% 0-1.9) | 0 (0% 0-4.3) |
| HPV40 | 21 (2.5% 1.5-3.7) | 5 (4.1% 1.4-9.4) | 0 (0% 0-1.9) | 1 (1.2% 0-6.5) |
| HPV42 | 38 (4.4% 3.2-6.1) | 7 (5.8% 2.4-11.6) | 5 (2.6% 0.8-5.9) | 0 (0% 0-4.3) |
| HPV43 | 17 (2% 1.2-3.2) | 3 (2.5% 0.5-7.1) | 1 (0.5% 0-2.8) | 1 (1.2% 0-6.5) |
| HPV44 | 20 (2.3% 1.4-3.6) | 1 (0.8% 0-4.5) | 5 (2.6% 0.8-5.9) | 0 (0% 0-4.3) |
| HPV54 | 38 (4.4% 3.2-6.1) | 5 (4.1% 1.4-9.4) | 4 (2.1% 0.6-5.2) | 0 (0% 0-4.3) |
| HPV55 | 13 (1.5% 0.8-2.6) | 0 (0% 0-3) | 0 (0% 0-1.9) | 1 (1.2% 0-6.5) |
| HPV57 | 0 (0% 0-0.4) | 0 (0% 0-3) | 0 (0% 0-1.9) | 0 (0% 0-4.3) |
| HPV61 | 8 (0.9% 0.4-1.8) | 1 (0.8% 0-4.5) | 1 (0.5% 0-2.8) | 0 (0% 0-4.3) |
| HPV71 | 0 (0% 0-0.4) | 0 (0% 0-3) | 0 (0% 0-1.9) | 0 (0% 0-4.3) |
| HPV72 | 1 (0.1% 0-0.7) | 0 (0% 0-3) | 0 (0% 0-1.9) | 0 (0% 0-4.3) |
| HPV81 | 23 (2.7% 1.7-4) | 0 (0% 0-3) | 3 (1.5% 0.3-4.5) | 0 (0% 0-4.3) |
| HPV83 | 18 (2.1% 1.3-3.3) | 3 (2.5% 0.5-7.1) | 1 (0.5% 0-2.8) | 0 (0% 0-4.3) |
| HPV84 | 19 (2.2% 1.3-3.5) | 2 (1.7% 0.2-5.8) | 3 (1.5% 0.3-4.5) | 0 (0% 0-4.3) |
| HPV89 | 6 (0.7% 0.3-1.5) | 1 (0.8% 0-4.5) | 1 (0.5% 0-2.8) | 0 (0% 0-4.3) |
|  |  |  |  |  |

**Table S2.**. **Prevalence of possibly carcinogenic and low risk (LR)-HPV genotype infections within histological diagnoses in HPV screened positive women.** Number of positive participants, prevalence (%) and 95% confidence intervals shown within each histological group. Results shown for individual HPV genotypes (irrespective of positivity for other genotypes).
