# Supplementary material for: Distribution of human papillomavirus genotypes by severity of cervical lesions in HPV screened positive women from the ESTAMPA study in Latin America
Source: PLoS One. 2022 Jul 29;17(7):e0272205. doi: 10.1371/journal.pone.0272205 (PMC9337688; doi:10.1371/journal.pone.0272205)
Supplement: S3 Table — Number of positive participants, prevalence (%) and 95% confidence intervals shown within each histological group. Results shown for individual HPV genotypes (irrespective of positivity for other genotypes). (DOCX) [file pone.0272205.s003.docx]

|  | ≤CIN1  n (% 95%IC)  n=399 | CIN2  n (% 95%IC)  n=68 | CIN3  n (% 95%IC)  n=124 | Cancer  n (% 95%IC)  n=75 |
| --- | --- | --- | --- | --- |
| HR-HPV genotypes (Group 1) |  |  |  |  |
| HPV16 | 54 (13.5% 10.3-17.3) | 12 (17.6% 9.5-28.8) | 68 (54.8% 45.7-63.8) | 50 (66.7% 54.8-77.1) |
| HPV18 | 23 (5.8% 3.7-8.5) | 7 (10.3% 4.2-20.1) | 4 (3.2% 0.9-8.1) | 5 (6.7% 2.2-14.9) |
| HPV31 | 29 (7.3% 4.9-10.3) | 6 (8.8% 3.3-18.2) | 15 (12.1% 6.9-19.2) | 1 (1.3% 0-7.2) |
| HPV33 | 12 (3% 1.6-5.2) | 1 (1.5% 0-7.9) | 9 (7.3% 3.4-13.3) | 0 (0% 0-4.8) |
| HPV35 | 13 (3.3% 1.7-5.5) | 4 (5.9% 1.6-14.4) | 4 (3.2% 0.9-8.1) | 0 (0% 0-4.8) |
| HPV39 | 24 (6% 3.9-8.8) | 2 (2.9% 0.4-10.2) | 4 (3.2% 0.9-8.1) | 1 (1.3% 0-7.2) |
| HPV45 | 20 (5% 3.1-7.6) | 2 (2.9% 0.4-10.2) | 1 (0.8% 0-4.4) | 7 (9.3% 3.8-18.3) |
| HPV51 | 20 (5% 3.1-7.6) | 2 (2.9% 0.4-10.2) | 1 (0.8% 0-4.4) | 2 (2.7% 0.3-9.3) |
| HPV52 | 36 (9% 6.4-12.3) | 9 (13.2% 6.2-23.6) | 5 (4% 1.3-9.2) | 4 (5.3% 1.5-13.1) |
| HPV56 | 34 (8.5% 6-11.7) | 3 (4.4% 0.9-12.4) | 2 (1.6% 0.2-5.7) | 0 (0% 0-4.8) |
| HPV58 | 18 (4.5% 2.7-7) | 5 (7.4% 2.4-16.3) | 8 (6.5% 2.8-12.3) | 2 (2.7% 0.3-9.3) |
| HPV59 | 31 (7.8% 5.3-10.8) | 3 (4.4% 0.9-12.4) | 0 (0% 0-2.9) | 1 (1.3% 0-7.2) |
| Probably HR-HPV genotype (Group2A)  HPV68 | 13 (3.3% 1.7-5.5) | 0 (0% 0-5.3) | 0 (0% 0-2.9) | 0 (0% 0-4.8) |
| Possibly HR-HPV genotype (Group2B) |  |  |  |  |
| HPV66 | 25 (6.3% 4.1-9.1) | 0 (0% 0-5.3) | 0 (0% 0-2.9) | 0 (0% 0-4.8) |
|  |  |  |  |  |

**Table S3**. **Prevalence of high-risk (HR) HPV genotype single infections within histological diagnoses in HPV screened positive women.** Number of positive participants, prevalence (%) and 95% confidence intervals shown within each histological group. Results shown for individual HPV genotypes (irrespective of positivity for other genotypes).
