# Supplementary material for: Distribution of human papillomavirus genotypes by severity of cervical lesions in HPV screened positive women from the ESTAMPA study in Latin America
Source: PLoS One. 2022 Jul 29;17(7):e0272205. doi: 10.1371/journal.pone.0272205 (PMC9337688; doi:10.1371/journal.pone.0272205)
Supplement: S4 Table — Number of participants, prevalence (%) and 95% confidence intervals shown within each histological group for all possible pairs (91 pairs) of HR genotypes ordered by their overall detection rate. (DOCX) [file pone.0272205.s004.docx]

|  | ≤CIN1 n=854  n (% 95%IC) | CIN2 n=121  n (% 95%IC) | CIN3 n=194  n (% 95%IC) | Cancer n=83  n (% 95%IC) |
| --- | --- | --- | --- | --- |
| HR-HPV genotypes pairs |  |  |  |  |
| HPV16+HPV31 | 10 (1.2% 0.6-2.1) | 3 (2.5% 0.5-7.1) | 4 (2.1% 0.6-5.2) | 0 (0% 0-4.3) |
| HPV16+HPV52 | 5 (0.6% 0.2-1.4) | 1 (0.8% 0-4.5) | 7 (3.6% 1.5-7.3) | 0 (0% 0-4.3) |
| HPV18+HPV52 | 7 (0.8% 0.3-1.7) | 2 (1.7% 0.2-5.8) | 3 (1.5% 0.3-4.5) | 0 (0% 0-4.3) |
| HPV31+HPV52 | 8 (0.9% 0.4-1.8) | 3 (2.5% 0.5-7.1) | 1 (0.5% 0-2.8) | 0 (0% 0-4.3) |
| HPV16+HPV39 | 6 (0.7% 0.3-1.5) | 1 (0.8% 0-4.5) | 4 (2.1% 0.6-5.2) | 0 (0% 0-4.3) |
| HPV16+HPV18 | 4 (0.5% 0.1-1.2) | 1 (0.8% 0-4.5) | 4 (2.1% 0.6-5.2) | 1 (1.2% 0-6.5) |
| HPV16+HPV66 | 7 (0.8% 0.3-1.7) | 0 (0% 0-3) | 2 (1% 0.1-3.7) | 1 (1.2% 0-6.5) |
| HPV52+HPV56 | 8 (0.9% 0.4-1.8) | 0 (0% 0-3) | 2 (1% 0.1-3.7) | 0 (0% 0-4.3) |
| HPV16+HPV58 | 5 (0.6% 0.2-1.4) | 1 (0.8% 0-4.5) | 2 (1% 0.1-3.7) | 1 (1.2% 0-6.5) |
| HPV16+HPV59 | 5 (0.6% 0.2-1.4) | 2 (1.7% 0.2-5.8) | 2 (1% 0.1-3.7) | 0 (0% 0-4.3) |
| HPV33+HPV52 | 8 (0.9% 0.4-1.8) | 0 (0% 0-3) | 1 (0.5% 0-2.8) | 0 (0% 0-4.3) |
| HPV51+HPV52 | 6 (0.7% 0.3-1.5) | 1 (0.8% 0-4.5) | 2 (1% 0.1-3.7) | 0 (0% 0-4.3) |
| HPV16+HPV56 | 3 (0.4% 0.1-1) | 0 (0% 0-3) | 5 (2.6% 0.8-5.9) | 0 (0% 0-4.3) |
| HPV31+HPV56 | 8 (0.9% 0.4-1.8) | 0 (0% 0-3) | 0 (0% 0-1.9) | 0 (0% 0-4.3) |
| HPV39+HPV52 | 4 (0.5% 0.1-1.2) | 1 (0.8% 0-4.5) | 3 (1.5% 0.3-4.5) | 0 (0% 0-4.3) |
| HPV45+HPV52 | 5 (0.6% 0.2-1.4) | 1 (0.8% 0-4.5) | 1 (0.5% 0-2.8) | 0 (0% 0-4.3) |
| HPV52+HPV58 | 7 (0.8% 0.3-1.7) | 0 (0% 0-3) | 0 (0% 0-1.9) | 0 (0% 0-4.3) |
| HPV52+HPV66 | 5 (0.6% 0.2-1.4) | 0 (0% 0-3) | 2 (1% 0.1-3.7) | 0 (0% 0-4.3) |
| HPV56+HPV59 | 7 (0.8% 0.3-1.7) | 0 (0% 0-3) | 0 (0% 0-1.9) | 0 (0% 0-4.3) |
| HPV16+HPV45 | 4 (0.5% 0.1-1.2) | 0 (0% 0-3) | 2 (1% 0.1-3.7) | 0 (0% 0-4.3) |
| HPV16+HPV51 | 4 (0.5% 0.1-1.2) | 0 (0% 0-3) | 2 (1% 0.1-3.7) | 0 (0% 0-4.3) |
| HPV31+HPV59 | 4 (0.5% 0.1-1.2) | 1 (0.8% 0-4.5) | 1 (0.5% 0-2.8) | 0 (0% 0-4.3) |
| HPV33+HPV56 | 5 (0.6% 0.2-1.4) | 0 (0% 0-3) | 1 (0.5% 0-2.8) | 0 (0% 0-4.3) |
| HPV39+HPV58 | 3 (0.4% 0.1-1) | 2 (1.7% 0.2-5.8) | 1 (0.5% 0-2.8) | 0 (0% 0-4.3) |
| HPV45+HPV51 | 6 (0.7% 0.3-1.5) | 0 (0% 0-3) | 0 (0% 0-1.9) | 0 (0% 0-4.3) |
| HPV51+HPV56 | 4 (0.5% 0.1-1.2) | 0 (0% 0-3) | 2 (1% 0.1-3.7) | 0 (0% 0-4.3) |
| HPV16+HPV68 | 3 (0.4% 0.1-1) | 1 (0.8% 0-4.5) | 1 (0.5% 0-2.8) | 0 (0% 0-4.3) |
| HPV18+HPV58 | 4 (0.5% 0.1-1.2) | 0 (0% 0-3) | 1 (0.5% 0-2.8) | 0 (0% 0-4.3) |
| HPV18+HPV66 | 3 (0.4% 0.1-1) | 1 (0.8% 0-4.5) | 0 (0% 0-1.9) | 1 (1.2% 0-6.5) |
| HPV39+HPV56 | 5 (0.6% 0.2-1.4) | 0 (0% 0-3) | 0 (0% 0-1.9) | 0 (0% 0-4.3) |
| HPV45+HPV58 | 2 (0.2% 0-0.8) | 1 (0.8% 0-4.5) | 2 (1% 0.1-3.7) | 0 (0% 0-4.3) |
| HPV56+HPV66 | 5 (0.6% 0.2-1.4) | 0 (0% 0-3) | 0 (0% 0-1.9) | 0 (0% 0-4.3) |
| HPV16+HPV33 | 2 (0.2% 0-0.8) | 1 (0.8% 0-4.5) | 0 (0% 0-1.9) | 1 (1.2% 0-6.5) |
| HPV16+HPV35 | 3 (0.4% 0.1-1) | 1 (0.8% 0-4.5) | 0 (0% 0-1.9) | 0 (0% 0-4.3) |
| HPV18+HPV31 | 2 (0.2% 0-0.8) | 1 (0.8% 0-4.5) | 1 (0.5% 0-2.8) | 0 (0% 0-4.3) |
| HPV18+HPV35 | 1 (0.1% 0-0.7) | 2 (1.7% 0.2-5.8) | 1 (0.5% 0-2.8) | 0 (0% 0-4.3) |
| HPV18+HPV45 | 2 (0.2% 0-0.8) | 1 (0.8% 0-4.5) | 1 (0.5% 0-2.8) | 0 (0% 0-4.3) |
| HPV18+HPV68 | 2 (0.2% 0-0.8) | 1 (0.8% 0-4.5) | 1 (0.5% 0-2.8) | 0 (0% 0-4.3) |
| HPV31+HPV33 | 2 (0.2% 0-0.8) | 1 (0.8% 0-4.5) | 0 (0% 0-1.9) | 1 (1.2% 0-6.5) |
| HPV31+HPV39 | 3 (0.4% 0.1-1) | 1 (0.8% 0-4.5) | 0 (0% 0-1.9) | 0 (0% 0-4.3) |
| HPV31+HPV45 | 3 (0.4% 0.1-1) | 1 (0.8% 0-4.5) | 0 (0% 0-1.9) | 0 (0% 0-4.3) |
| HPV31+HPV51 | 4 (0.5% 0.1-1.2) | 0 (0% 0-3) | 0 (0% 0-1.9) | 0 (0% 0-4.3) |
| HPV31+HPV58 | 4 (0.5% 0.1-1.2) | 0 (0% 0-3) | 0 (0% 0-1.9) | 0 (0% 0-4.3) |
| HPV33+HPV58 | 2 (0.2% 0-0.8) | 1 (0.8% 0-4.5) | 1 (0.5% 0-2.8) | 0 (0% 0-4.3) |
| HPV35+HPV45 | 3 (0.4% 0.1-1) | 1 (0.8% 0-4.5) | 0 (0% 0-1.9) | 0 (0% 0-4.3) |
| HPV35+HPV52 | 4 (0.5% 0.1-1.2) | 0 (0% 0-3) | 0 (0% 0-1.9) | 0 (0% 0-4.3) |
| HPV35+HPV59 | 4 (0.5% 0.1-1.2) | 0 (0% 0-3) | 0 (0% 0-1.9) | 0 (0% 0-4.3) |
| HPV39+HPV51 | 2 (0.2% 0-0.8) | 1 (0.8% 0-4.5) | 1 (0.5% 0-2.8) | 0 (0% 0-4.3) |
| HPV45+HPV56 | 2 (0.2% 0-0.8) | 0 (0% 0-3) | 2 (1% 0.1-3.7) | 0 (0% 0-4.3) |
| HPV45+HPV59 | 4 (0.5% 0.1-1.2) | 0 (0% 0-3) | 0 (0% 0-1.9) | 0 (0% 0-4.3) |
| HPV51+HPV58 | 4 (0.5% 0.1-1.2) | 0 (0% 0-3) | 0 (0% 0-1.9) | 0 (0% 0-4.3) |
| HPV51+HPV59 | 3 (0.4% 0.1-1) | 1 (0.8% 0-4.5) | 0 (0% 0-1.9) | 0 (0% 0-4.3) |
| HPV51+HPV68 | 2 (0.2% 0-0.8) | 2 (1.7% 0.2-5.8) | 0 (0% 0-1.9) | 0 (0% 0-4.3) |
| HPV51+HPV66 | 3 (0.4% 0.1-1) | 1 (0.8% 0-4.5) | 0 (0% 0-1.9) | 0 (0% 0-4.3) |
| HPV56+HPV68 | 4 (0.5% 0.1-1.2) | 0 (0% 0-3) | 0 (0% 0-1.9) | 0 (0% 0-4.3) |
| HPV58+HPV59 | 3 (0.4% 0.1-1) | 1 (0.8% 0-4.5) | 0 (0% 0-1.9) | 0 (0% 0-4.3) |
| HPV18+HPV51 | 3 (0.4% 0.1-1) | 0 (0% 0-3) | 0 (0% 0-1.9) | 0 (0% 0-4.3) |
| HPV18+HPV56 | 2 (0.2% 0-0.8) | 0 (0% 0-3) | 1 (0.5% 0-2.8) | 0 (0% 0-4.3) |
| HPV31+HPV35 | 2 (0.2% 0-0.8) | 0 (0% 0-3) | 1 (0.5% 0-2.8) | 0 (0% 0-4.3) |
| HPV35+HPV58 | 2 (0.2% 0-0.8) | 1 (0.8% 0-4.5) | 0 (0% 0-1.9) | 0 (0% 0-4.3) |
| HPV39+HPV59 | 2 (0.2% 0-0.8) | 1 (0.8% 0-4.5) | 0 (0% 0-1.9) | 0 (0% 0-4.3) |
| HPV45+HPV66 | 3 (0.4% 0.1-1) | 0 (0% 0-3) | 0 (0% 0-1.9) | 0 (0% 0-4.3) |
| HPV52+HPV68 | 2 (0.2% 0-0.8) | 0 (0% 0-3) | 1 (0.5% 0-2.8) | 0 (0% 0-4.3) |
| HPV56+HPV58 | 2 (0.2% 0-0.8) | 0 (0% 0-3) | 1 (0.5% 0-2.8) | 0 (0% 0-4.3) |
| HPV18+HPV59 | 2 (0.2% 0-0.8) | 0 (0% 0-3) | 0 (0% 0-1.9) | 0 (0% 0-4.3) |
| HPV31+HPV68 | 1 (0.1% 0-0.7) | 0 (0% 0-3) | 1 (0.5% 0-2.8) | 0 (0% 0-4.3) |
| HPV31+HPV66 | 1 (0.1% 0-0.7) | 0 (0% 0-3) | 1 (0.5% 0-2.8) | 0 (0% 0-4.3) |
| HPV33+HPV39 | 1 (0.1% 0-0.7) | 0 (0% 0-3) | 1 (0.5% 0-2.8) | 0 (0% 0-4.3) |
| HPV33+HPV59 | 1 (0.1% 0-0.7) | 1 (0.8% 0-4.5) | 0 (0% 0-1.9) | 0 (0% 0-4.3) |
| HPV35+HPV51 | 1 (0.1% 0-0.7) | 1 (0.8% 0-4.5) | 0 (0% 0-1.9) | 0 (0% 0-4.3) |
| HPV35+HPV56 | 2 (0.2% 0-0.8) | 0 (0% 0-3) | 0 (0% 0-1.9) | 0 (0% 0-4.3) |
| HPV35+HPV68 | 1 (0.1% 0-0.7) | 1 (0.8% 0-4.5) | 0 (0% 0-1.9) | 0 (0% 0-4.3) |
| HPV39+HPV68 | 2 (0.2% 0-0.8) | 0 (0% 0-3) | 0 (0% 0-1.9) | 0 (0% 0-4.3) |
| HPV45+HPV68 | 2 (0.2% 0-0.8) | 0 (0% 0-3) | 0 (0% 0-1.9) | 0 (0% 0-4.3) |
| HPV58+HPV68 | 2 (0.2% 0-0.8) | 0 (0% 0-3) | 0 (0% 0-1.9) | 0 (0% 0-4.3) |
| HPV59+HPV68 | 0 (0% 0-0.4) | 0 (0% 0-3) | 1 (0.5% 0-2.8) | 1 (1.2% 0-6.5) |
| HPV59+HPV66 | 2 (0.2% 0-0.8) | 0 (0% 0-3) | 0 (0% 0-1.9) | 0 (0% 0-4.3) |
| HPV68+HPV66 | 1 (0.1% 0-0.7) | 1 (0.8% 0-4.5) | 0 (0% 0-1.9) | 0 (0% 0-4.3) |
| HPV18+HPV39 | 1 (0.1% 0-0.7) | 0 (0% 0-3) | 0 (0% 0-1.9) | 0 (0% 0-4.3) |
| HPV33+HPV35 | 1 (0.1% 0-0.7) | 0 (0% 0-3) | 0 (0% 0-1.9) | 0 (0% 0-4.3) |
| HPV33+HPV51 | 0 (0% 0-0.4) | 0 (0% 0-3) | 1 (0.5% 0-2.8) | 0 (0% 0-4.3) |
| HPV33+HPV68 | 1 (0.1% 0-0.7) | 0 (0% 0-3) | 0 (0% 0-1.9) | 0 (0% 0-4.3) |
| HPV33+HPV66 | 0 (0% 0-0.4) | 0 (0% 0-3) | 1 (0.5% 0-2.8) | 0 (0% 0-4.3) |
| HPV35+HPV39 | 1 (0.1% 0-0.7) | 0 (0% 0-3) | 0 (0% 0-1.9) | 0 (0% 0-4.3) |
| HPV35+HPV66 | 0 (0% 0-0.4) | 1 (0.8% 0-4.5) | 0 (0% 0-1.9) | 0 (0% 0-4.3) |
| HPV39+HPV45 | 1 (0.1% 0-0.7) | 0 (0% 0-3) | 0 (0% 0-1.9) | 0 (0% 0-4.3) |
| HPV39+HPV66 | 1 (0.1% 0-0.7) | 0 (0% 0-3) | 0 (0% 0-1.9) | 0 (0% 0-4.3) |
| HPV52+HPV59 | 1 (0.1% 0-0.7) | 0 (0% 0-3) | 0 (0% 0-1.9) | 0 (0% 0-4.3) |
| HPV58+HPV66 | 1 (0.1% 0-0.7) | 0 (0% 0-3) | 0 (0% 0-1.9) | 0 (0% 0-4.3) |
| HPV18+HPV33 | 0 (0% 0-0.4) | 0 (0% 0-3) | 0 (0% 0-1.9) | 0 (0% 0-4.3) |
| HPV33+HPV45 | 0 (0% 0-0.4) | 0 (0% 0-3) | 0 (0% 0-1.9) | 0 (0% 0-4.3) |
|  |  |  |  |  |

**Table S4. Prevalence of high-risk (HR)-HPV genotype double infections within histological diagnoses in HPV screened positive women.** Number of participants, prevalence (%) and 95% confidence intervals shown within each histological group for all possible pairs (91 pairs) of HR genotypes ordered by their overall detection rate.
