# Supplementary material for: Distribution of human papillomavirus genotypes by severity of cervical lesions in HPV screened positive women from the ESTAMPA study in Latin America
Source: PLoS One. 2022 Jul 29;17(7):e0272205. doi: 10.1371/journal.pone.0272205 (PMC9337688; doi:10.1371/journal.pone.0272205)
Supplement: S5 Table — Number of positive participants and prevalence (%) for each genotype shown within each histological diagnosis. Risk ratio (RR) with 95% confidence interval shown considering as reference group women aged 30–39. Risk ratios and confidence intervals not computed in case of null frequencies. P-values from a trend test for proportions shown. (DOCX) [file pone.0272205.s005.docx]

|  |  | HPV18 | | HPV31 | | HPV45 | | HPV52 | | |
| --- | --- | --- | --- | --- | --- | --- | --- | --- | --- | --- |
|  | n | n(%) | RR (95%CI) | n(%) | RR (95%CI) | n(%) | RR (95%CI) | n(%) | RR (95%CI) | |
|  |  |  |  |  |  |  |  |  |  | |
| ≤CIN1 |  |  |  |  |  |  |  |  |  | |
| All | 854 | 51(6%) |  | 88 (10.3%) |  | 50 (5.9%) |  | 95 (11.1%) |  | |
| 30-39 | 292 | 19 (5.6%) | 1 | 44 (13.1%) | 1 | 26 (7.7%) | 1 | 43 (12.8%) | | 1 |
| 40-49 | 212 | 14 (5.6%) | 0.99 (0.51-1.93) | 19 (7.6%) | 0.58 (0.35-0.97) | 9 (3.6%) | 0.46 (0.22-0.97) | 26 (10.4%) | | 0.81 (0.51-1.28) |
| 50-65 | 224 | 18 (6.8%) | 1.2 (0.64-2.24) | 25 (9.4%) | 0.72 (0.45-1.14) | 15 (5.6%) | 0.73 (0.4-1.35) | 26 (9.8%) | | 0.77 (0.48-1.21) |
|  |  |  |  |  |  |  |  |  | |  |
| CIN2 |  |  |  |  |  |  |  |  | |  |
| All | 121 | 14 (11.6%) |  | 14 (11.6%) |  | 5 (4.1%) |  | 19 (15.7%) | |  |
| 30-39 | 73 | 8 (10.7%) | 1 | 9 (12%) | 1 | 2 (2.7%) | 1 | 15 (20%) | | 1 |
| 40-49 | 28 | 4 (13.8%) | 1.29 (0.42-3.97) | 5 (17.2%) | 1.44 (0.53-3.93) | 1 (3.4%) | 1.29 (0.12-13.72) | 1 (3.4%) | | 0.17 (0.02-1.25) |
| 50-65 | 14 | 2 (11.8%) | 1.1 (0.26-4.74) | 0 (0%) | - | 2 (11.8%) | 4.41 (0.67-29.14) | 3 (17.6%) | | 0.88 (0.29-2.71) |
|  |  |  |  |  |  |  |  |  | |  |
| CIN3 |  |  |  |  |  |  |  |  | |  |
| All | 194 | 14 (7.2%) |  | 25 (12.9%) |  | 5 (2.6%) |  | 22 (11.3%) | |  |
| 30-39 | 103 | 9 (8.5%) | 1 | 16 (15.1%) | 1 | 3 (2.8%) | 1 | 14 (13.2%) | | 1 |
| 40-49 | 54 | 2 (3.6%) | 0.42 (0.09-1.88) | 7 (12.5%) | 0.83 (0.36-1.89) | 2 (3.6%) | 1.26 (0.22-7.33) | 6 (10.7%) | | 0.81 (0.33-2) |
| 50-65 | 31 | 3 (9.4%) | 1.1 (0.32-3.84) | 2 (6.2%) | 0.41 (0.1-1.71) | 0 (0%) | - | 2 (6.2%) | | 0.47 (0.11-1.97) |
|  |  |  |  |  |  |  |  |  | |  |
| Cancer |  |  |  |  |  |  |  |  | |  |
| All | 83 | 6 (7.2%) |  | 3 (3.6%) |  | 7 (8.4%) |  | 4 (4.8%) | |  |
| 30-39 | 22 | 2 (9.1%) | 1 | 2 (9.1%) | 1 | 1 (4.5%) | 1 | 0 (0%) | | - |
| 40-49 | 28 | 3 (10.7%) | 1.18 (0.22-6.45) | 0 (0%) | - | 4 (14.3%) | 3.14 (0.38-26.16) | 0 (0%) | | - |
| 50-65 | 33 | 1 (3%) | 0.33 (0.03-3.46) | 1 (3%) | 0.33 (0.03-3.46) | 2 (6.1%) | 1.33 (0.13-13.83) | 4 (12.1%) | - | |
|  |  |  |  |  |  |  |  |  |  | |
|  |  |  |  |  |  |  |  |  |  | |

**Table S5.** **Prevalence of HPV18, HPV31, HPV45 and HPV52 genotypes within histological diagnoses overall and by age in HPV screened positive women.** Number of positive participants and prevalence (%) for each genotype shown within each histological diagnosis. Risk ratio (RR) with 95% confidence interval shown considering as reference group women aged 30-39. Risk ratios and confidence intervals not computed in case of null frequencies. P-values from a trend test for proportions shown.
